# Radiomic tractometry: a rich and tract-specific class of imaging biomarkers for neuroscience and medical applications

**DOI:** 10.21203/rs.3.rs-2950610/v1

**Published:** 2023-05-23

**Authors:** Peter Neher, Dusan Hirjak, Klaus Maier-Hein

**Affiliations:** Division of Medical Image Computing, German Cancer Research Center, Heidelberg, Germany; Department of Psychiatry and Psychotherapy, Central Institute of Mental Health, Medical Faculty Mannheim, Heidelberg University, Mannheim, Germany; Division of Medical Image Computing, German Cancer Research Center, Heidelberg, Germany

## Abstract

Tract-specific microstructural analysis of the brain white matter using diffusion MRI is a driver for neuroscientific discovery with a wide range of applications. Current analysis pipelines have conceptual limitations that narrow their applicability and hamper subject-level analysis and predictions.

Radiomic tractometry (RadTract) improves upon that, enabling the extraction and analysis of comprehensive and highly informative microstructural feature sets where previous approaches were restricted to bare summary statistics. We demonstrate the added value in a series of neuroscientific applications, including diagnostic tasks as well as the prediction of demographic and clinical measures across various datasets.

Being published as an open and easy-to-use Python package, RadTract could spark the establishment of a new generation of tract-specific imaging biomarkers, with direct benefits for a range of applications from basic neuroscience to medical research.

## Main

A key element in understanding healthy and impaired brain structure and function is the analysis of its neural pathways, the white matter (WM). Over the last decades, the development of diffusion-weighted magnetic resonance imaging (dMRI) has revolutionized our ability to study WM in vivo. By probing the movement of water molecules, dMRI provides information about the microstructure, integrity, and connectivity of WM tracts. Many highly influential studies based on dMRI have been published, analyzing the WM and its alterations to gain insights into brain development, aging, injuries, and diseases or to study normal brain structure and function^1–8^.

Analysis of brain-MRI data has progressed from global histogram-based analysis^9,10^, over voxel-based statistical analysis matching individual subjects using registration algorithms^11,12^, to WM-skeleton-based analysis of whole-brain data using tract-based spatial statistics (TBSS)^13^. While used frequently, these techniques have several limitations discussed extensively in the literature^14,13,15^. Recently, there has been a shift towards tract-specific approaches based on fiber tractography^16–18^. These methods enable the targeted investigation of WM microstructure, in the form of dMRI-derived parameter maps such as the fractional anisotropy (FA) or mean diffusivity (MD)^19^, within specific tracts.

Tract-specific analysis itself has evolved from studying tract averages^20,17,18^ to an analysis of microstructural parameters along individual tracts^21–24^. Tract averages involve the calculation of statistics over the entire tract, which can be useful for investigating global changes in tract integrity. However, it does not provide information about regional variations within the tract, which are quite significant. In contrast, along-tract analysis (tractometry) involves dividing the tract into smaller parcels along its course and analyzing the diffusion metrics in each parcel separately. This method provides a detailed picture of variations within the tract, allowing for the investigation of localized tissue alterations and specific functions associated with different parts of the tract. Tractometry has been used extensively in a variety of applications and can be considered as the state-of-the-art in tract-specific WM analysis^24–33^.

Nevertheless, tractometry as well as other techniques widely used in neuroscience such as voxel-based analysis or TBSS are designed for group-level statistical analysis and only allow limited or no statements at the individual subject level. To enable subject-level analysis and prediction, conceptual as well as technical aspects of tractometry need to be reconsidered with a focus on leveraging the full potential of the underlying dMRI data for advanced analysis methods based on machine learning (ML).

Tractometry is based on models of individual WM tracts obtained using fiber tractography^16^. The tract models consist of individual fibers, or streamlines, each of which is a series of 3D points that define its trajectory. To analyze the image along the tract, it is evaluated at these points and each value is assigned to one of *n* parcels depending on its position. There are two main approaches for parcel assignment: one statically resamples the streamlines to *n* points (1)^23^, and the other assigns each value at a streamline point to the closest point on a tract-centerline composed of *n* points (2)^34–36^. The values within each parcel are then aggregated, usually by averaging, resulting in a vector of scalar values along the tract. This tract profile can then be used for further analysis.

**Figure 1:**
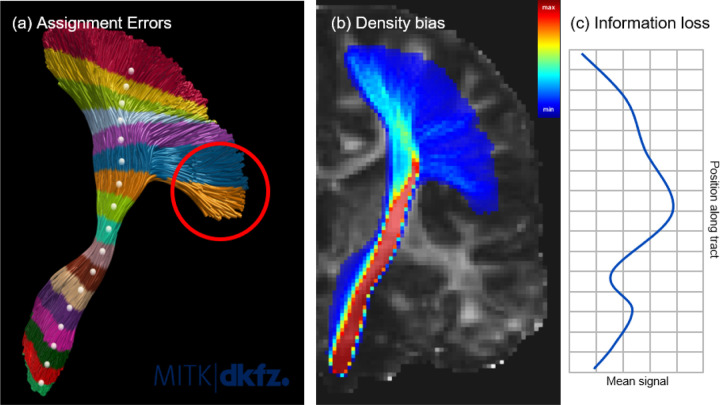
Illustration of the issues classic tractometry is struggling with: assignment of tract parts, here of the corticospinal tract (CST), to wrong parcels using the centerline approach (a), intra-tract density variations leading to a bias towards the core parts of the tract (b), and a loss of information due to the reduction of the complete image information visible in (b) along the tract to a few tract profile values (c).

On a technical level, this approach suffers from assignment errors between tract positions and parcels. For assignment strategy (1), these errors arise from misalignment among the individual streamlines, causing image values at the same spatial position to be assigned to different parcels based on their respective positions along the streamline. To mitigate this, the current practice is to truncate the entire tract to a central part by excluding fanning start and end regions. However, this eliminates entire regions of the tract from further analysis. In the case of assignment strategy (2), a single centerline is insufficient to represent the entire tract. Particularly in tracts with a lot of fanning, this leads to assignment errors (Fig. 1a).

On a conceptual level, tractometry suffers from a bias towards image values located in dense tract regions close to the tract centerlines. This is because tractometry works in streamline- and not voxel-space. Consequently, regions located closer to the tract margins, as well as near the endings where the tract fans out towards the cortex, are often undersampled by factors larger than 100 compared to the tract-core (Fig. 1b). Another issue is the potential loss of information due to the reduction of the complete image information along the tract to a few tract profile values (Fig. 1c).

We developed a new approach, radiomic tractometry (RadTract), that avoids wrong parcel assignments, has no density bias by operating directly in voxel-space, and leverages the full richness of the image data by calculating a comprehensive set of statistical, shape, and texture features along the tract. RadTract provides an improved localization of WM alterations and is much more sensitive to microstructural changes compared to classic tractometry.

We demonstrate these capabilities and RadTract’s general applicability on four distinct datasets comprising individuals with Alzheimer’s disease, Mild Cognitive Impairment, Parkinson’s disease, prodromal Parkinson’s disease, schizophrenia, and catatonia as well as matched healthy controls (HC). RadTract significantly outperforms classic tractometry in diagnosing disease subgroups in all datasets. Using RadTract it is further possible to estimate demographic and clinical parameters on a subject level, such as age, education, symptom severity, or daily medication dose, with a much better correlation and also in multiple tracts where this is not possible using classic tractometry.

Overall, our results indicate that even well-studied parameter maps, such as the FA, contain a wealth of information that could be extremely valuable for a wide range of applications including subject-level analyses, but that is currently lost for the state-of-the-art.

We anticipate this work to be a starting point for the development of a new generation of tract-specific imaging biomarkers, enabling not only better neuroscientific studies on a subject level but with a direct benefit for patients and clinicians. In the future, RadTract-based features could be used as biomarkers of clinical symptoms and outcomes, physical fitness, and global as well as cognitive functioning in patients and healthy subjects. In addition, we expect RadTract to spare critically ill and non-adherent psychiatric and neurological patients from lengthy diagnostic tests that are tedious, difficult to perform, and expensive. Going beyond medical applications, RadTract has the potential to further our understanding of the biological processes involved in the development and aging of the human brain by providing a much more detailed and structured view on microstructural patterns and their changes. Our approach is also not limited to dMRI and WM analysis, but it is generally applicable to all kinds of imaging contrasts and also to all kinds of research questions involving fibrous tissue and tractography thereof, such as the analysis of microstructural properties of muscular tissue or the neurovascular anatomy of the prostate^37,38^.

RadTract is available as an easy-to-use open-source Python package.

## Results

We present results obtained on four datasets, including data obtained from the Alzheimer’s Disease Neuroimaging Initiative (ADNI, www.adni-info.org/), the Parkinson’s Progression Markers Initiative (PPMI, www.ppmi-info.org/access-data-specimens/download-data, RRID:SCR_006431), the UCLA Consortium for Neuropsychiatric Phenomics LA5c Study (SCHZ, https://openfmri.org/dataset/ds000030/)^39^ and a non-public dataset (CAT) acquired at the Central Institute of Mental Health (CIMH, https://www.zi-mannheim.de/en/)^27^. These distinct datasets enabled a broad range of experiments on imaging data of healthy and diseased individuals, well suited to demonstrate the capabilities and general applicability of our approach. A total of 46 WM tracts were investigated individually. Since it is the most exhaustively studied microstructural WM parameter, we chose the FA for our experiments and demonstrate that RadTract yields much more valuable information than previously possible even from this relatively basic parameter map. Please refer to the methods section for more details on the used datasets, preprocessing, and tract modeling.

As a baseline method, we used classic centerline-tractometry^27,36^, which is the state-of-the-art for tract-specific analysis. Other widely used techniques, such as voxel-based analysis or TBSS were not included as benchmarks, since they are designed for purely global group-level analysis and do not yield tract-specific features usable for subject-level predictions.

### RadTract parcellations show improved anatomical cohesion

RadTract parcellation was performed for all tracts and subjects. The number of parcels *n* for RadTract was automatically determined as described in the methods. The corresponding values can be found in Supplementary S1.1. To enable a direct comparison, a parcellation of each tract was further created using the centerline-based approach with the same number of parcels.

To quantify the differences between the proposed parcellation approach and the centerline-parcellation, a parcel-wise dice coefficient was calculated for all subjects in all datasets. As expected, in tightly packed tube-shaped parts of each tract, the two parcellations are relatively similar, which is reflected in moderate to high dice scores in these regions. Deviations are strongest in fanning areas, which is reflected in low dice scores. Plots showing the dice scores for all tracts and parcels can be found in Supplementary S1.2.

### RadTract features enable a rich representation of image information along tracts

**Figure 3:**
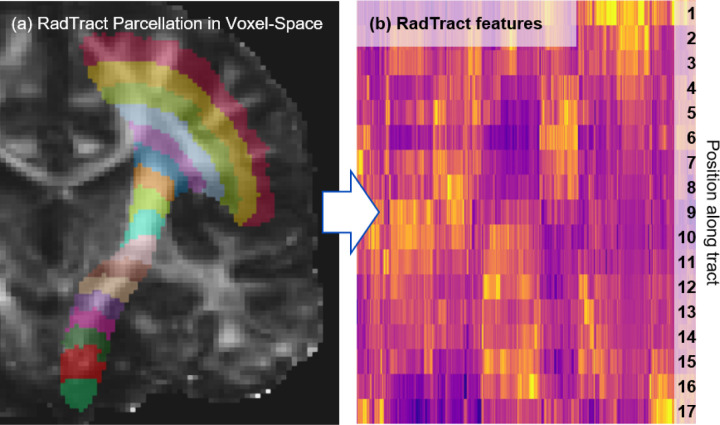
Illustration of the RadTract-based CST parcellation of an exemplary subject (a) and the corresponding features as a heatmap (b). Per line, i.e. parcel, all 1106 features are visualized.

Classic tractometry reduces the complete image information along the tract to a relatively small number of averages (100 for all subsequent experiments), as visualized in the introduction (Fig. 1). In contrast, RadTract leverages the well-established concept of radiomics for extracting as much information as possible from the image section covered by the respective tract of interest in the form of well-defined and standardized features^40,41^. We applied this approach to the individual parcels resulting from the proposed tract parcellation approach. For each parcel, RadTract yields a set of 105 features on the original image as well as 91 features on 11 filtered versions of the image, resulting in a total of 1106 features per parcel. Shape features were only calculated on the original image. Figure 3 provides a heatmap visualization of the features obtained from the CST of an exemplary subject as well as the corresponding parcellation. The complete list of features used in this work can be found in Supplementary S6. Please refer to the methods section for more information about the feature calculation and a visualization of the complete RadTract process.

This feature set served as a rich basis for the successive ML-based subject-level predictions and, in conjunction with the improved anatomical cohesion of the used parcellation, enabled a more accurate and valuable localization of tract regions relevant for the respective task.

### RadTract features enable improved automatic diagnoses

To analyze the value of RadTract features for automatic diagnosis, we performed classification experiments to identify patient subgroups with distinct diagnoses in all datasets. These subgroups not only comprised the classes “healthy” and “diseased” but different stages of the diseases, which made the task much more challenging but at the same time more relevant. Please refer to the methods section for a description of the datasets and subgroups. Classification experiments were performed on each dataset and for each tract individually. Each experiment was performed using centerline-tractometry features, all RadTract features as well as eleven subsets of the complete RadTract feature set (e.g., using only shape or only first-order intensity features). Automatic feature selection was performed to reduce the large number of RadTract features to a reasonable number, here 500. A list of all analyzed RadTract feature sets and details on the automatic feature selection can be found in the methods section. To obtain reliable performance indicators, each experiment was realized as ten times differently seeded leave-one-out cross-validation (LOO-CV), resulting in 5,980 experiments. We used a random forest with 500 trees and no further hyperparameter optimization for classification.

**Figure 4:**
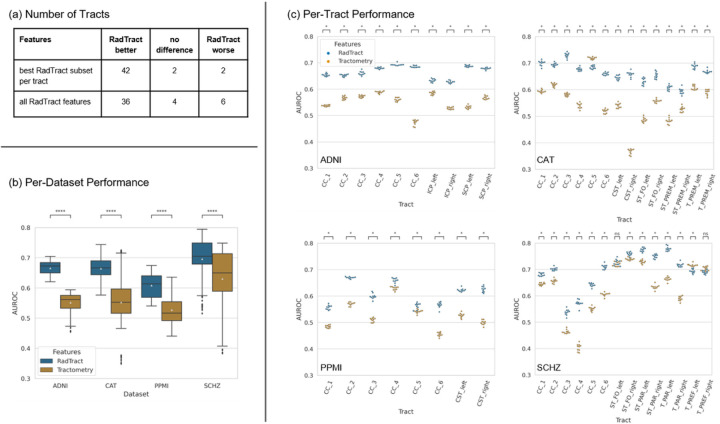
Classification results for all datasets using classic tractometry in comparison to RadTract. In (a) the number of tracts where RadTract features lead to better or worse mean classification results (statistically significant) compared to classic tractometry, for two different feature configurations. (b) shows the classification performance for all experiments per dataset. In (c) the classification results on each dataset and for each tract individually are shown. RadTract significantly outperforms classic tractometry by 11.4, 11.2, 8.1, and 6.5 points AUROC for the datasets ADNI, CAT, PPMI, and SCHZ respectively. The plotted results in (b) and (c) were obtained with the respective best feature subset per tract. The plots for all feature subsets, tracts, and datasets can be found in Supplementary S2.

Performance differences were tested for significance using a Wilcoxon signed-rank test and Bonferroni correction. RadTract features lead to improved results for all datasets, as illustrated in Fig. 4. RadTract significantly outperforms classic tractometry by 11.4, 11.2, 8.1, and 6.5 points AUROC for the best feature subset per tract in the datasets ADNI, CAT, PPMI, and SCHZ, respectively. It yields better results in 42 out of 46 tract/dataset combinations, results without significant difference in 2, and results with lower scores in 2 combinations. RadTract further yields very promising results in 18 tracts, where tractometry features only yield results close to the level of random guessing (AUROC < 0.55). The results for all feature subsets, tracts, and datasets can be found in Supplementary S2.

### RadTract features enable improved prediction of demographic and clinical parameters

To demonstrate the value of RadTract for tasks beyond automatic diagnosis and also beyond medical applications, we performed experiments to automatically predict demographic (age, number of pack-years, years of education) and clinical parameters (BPRS total, PANSS total, GAF scores, and olanzapine equivalents (OLZe)) on patients of the CAT dataset. A description of the individual parameters can be found in the methods.

The experiment setup was similar to the automatic diagnosis experiments but limited to the CAT datasets. Training and testing of the random forest regressor were performed with a fixed split instead of LOO-CV. This resulted in 12,740 regression experiments.

Performance differences were tested for significance using a Wilcoxon signed-rank test and Bonferroni correction. Pearson correlations were tested for significance using a two-sided t-test without correction for multiple comparisons.

**Figure 5:**
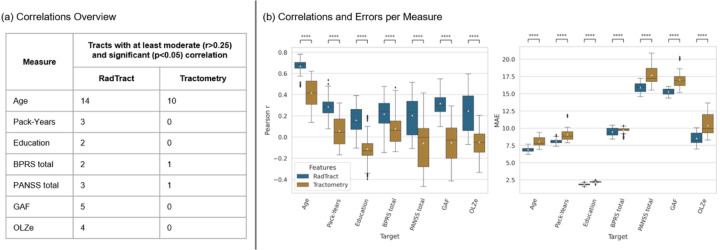
Regression results for seven demographic (Age, Pack-Years, Education) and clinical (BPRS total, PANSS total, GAF, OLZe) parameters. The numbers of tracts yielding at least moderate and statistically significant correlations between predicted and true measures for RadTract and centerline-tractometry are shown in (a). (b) shows all correlations (left) and mean absolute errors (right) across tracts as boxplots for each measure. All results were obtained with the respective best parameter subset per tract. The plots for all feature subsets and tracts can be found in Supplementary S3.

RadTract showed an on average much higher correlation of the predicted parameters with the ground truth as centerline-tractometry. RadTract showed significant correlations in seventeen tracts where this was not possible using classic tractometry. Remarkably, using classic tractometry no at least moderate (r > 0.25) and statistically significant correlation could be obtained for the parameters number of pack-years, years of education, GAF score, and OLZe, while RadTract yielded significant correlations in multiple tracts for all parameters. The mean absolute prediction error was reduced significantly when using RadTract for all parameters. See Fig. 5 for details. All results were obtained with the respective best parameter subset per tract. The results for all feature subsets and tracts can be found in Supplementary S3.

### RadTract features enable improved localization of important tract regions

RadTract allows a detailed analysis of the importance of different classes of features as well as of the different tract parcels, here demonstrated for the automatic diagnosis task. The importance of individual features is provided by the random forests in the form of the mean decrease in impurity introduced by each feature. The importance of a parcel was calculated as the sum of the individual feature importances of that parcel, averaged over LOO-CV folds and replicates. The following analysis was performed on the optimal feature subset configuration per parcel.

**Figure 6:**
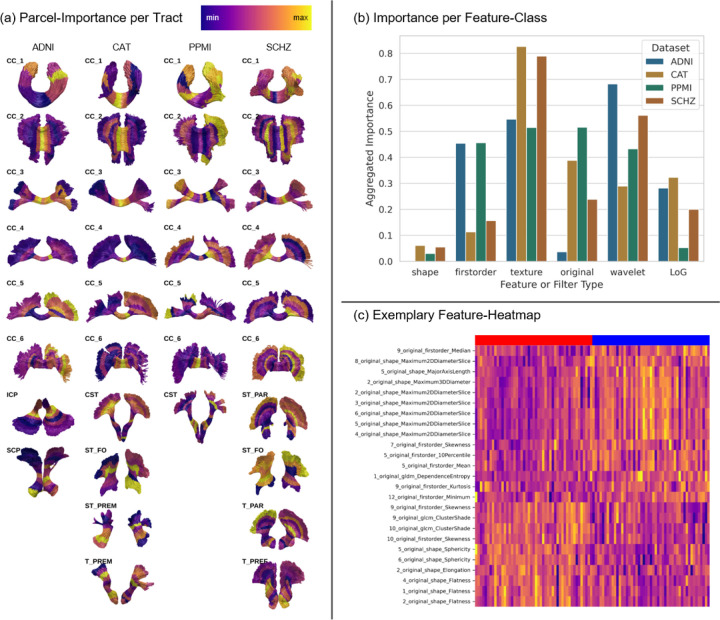
Parcel-importances for group separation projected onto the tracts of each dataset are shown in (a). The tracts are manually rotated along the y-axis for optimal visualization. In (b), the importance of different feature classes and features obtained with different image filters are shown. Values in (b) are only calculated for the CC tracts since these are analyzed in all datasets. An exemplary heatmap of the top-25 most important features obtained in the Thalamo-Parietal Tracts of the SCHZ dataset is shown in (c). The feature columns of the two subgroups are marked by a blue and a red bar, respectively, and can even be separated quite well visually.

Figure 6a illustrates the importance of each tract parcel for each task. In 14 out of 46 tract/dataset combinations, the tract start and/or end regions have a higher importance than the central 50% of the tract. This is interesting because these regions have low anatomical cohesion using the centerline approach, which makes correct localization of changes difficult.

Figure 6b illustrates the importance of different feature classes. Shape features played a minor role in all datasets, but seem to be more important in the neurodevelopmental (early-onset: SCHZ and CAT) compared to the neurodegenerative datasets (late-onset: ADNI and PPMI). In the former datasets, texture features were by far the most important, while in the latter, first-order and texture features played a similar role. No such clear pattern can be found for the importance of the different image filters.

## Discussion

We present radiomic tractometry (RadTract), a new approach for quantifying fibrous tissue such as the brain WM along its course. RadTract extends the state-of-the-art in tract-specific tissue analysis (tractometry) from a group-level analysis tool to subject-level prediction. This is achieved by computing a rich set of standardized first-order, shape, and texture features in contrast to the limited information provided by classical approaches (tractometry), and by enabling improved localization of important tract regions by providing tract parcellations with much better anatomical cohesion compared to the state-of-the-art.

We conducted a series of experiments in multiple datasets, illustrating the general applicability of RadTract as well as its superior performance compared to the state-of-the-art in various tasks. Our experiments show that RadTract is capable of extracting much more meaningful information from images than it is possible with classic tractometry, enabling new insights even when using well-studied and long-established parameter maps such as the FA. In general, RadTract supports arbitrary parameter maps besides the FA as well as other image contrasts as input, which, if chosen smartly for the respective task at hand, are expected to yield even more valuable features. While our experiments focused on ML and subject-level predictions, RadTract also enables a much more detailed classic statistical analysis, leveraging a whole range of features that provide information about the distribution of microstructural values beyond the typically analyzed first-order mean values.

Since RadTract yields numerous features, the issue of dataset size is quite important. While RadTract has already yielded many improvements, as shown in our experiments, its use in studies with larger samples is expected to lead to much better generalization capabilities. In this context, feature selection plays a crucial role. Using more advanced feature selection techniques on more training data could further improve the performance of RadTract features in the downstream tasks.

Our experiments further showed that the start- and end-regions of tracts, which are either completely discarded by existing approaches or that are not properly analyzable due to parcel-misassignments, do hold valuable information for subject-level predictions, and should therefore be analyzed as thoroughly as the central parts of the tracts. An as-of-yet open question is the impact of the exact size and shape of the individual parcels, which will be investigated in future studies. In this context, an aspect that should be addressed is the lack of a soundly defined reference for tract parcellations. On a tract level, multiple expert-annotated datasets have been published recently, but no such annotations exist on a parcel level. While our results convincingly demonstrate that RadTract parcellations avoid errors of state-of-the-art, quantitative evaluation of this aspect would be desirable. An investigation of potential manual parcellation references might also include the question of more meaningful parcel borders based on biologically-founded subdivisions of WM tracts.

In summary, RadTract defines a new state-of-the-art for tract-specific tissue analysis. We expect the presented work to be a starting point for a new generation of imaging biomarkers in the neuroscientific domain and beyond. We propose to leverage RadTract as an out-of-the-box tool for the calculation of advanced and standardized tract-specific imaging features, serving as a foundation for all kinds of custom-tailored applications in basic as well as applied research projects across research fields.

## Methods

In the following subsections, we will describe the data used in our experiments, the preprocessing of the data to generate the input for RadTract, the actual RadTract methodology itself, consisting of the tract parcellation and the feature calculation, as well as some implementation details of RadTract.

### Data and preprocessing steps

#### ADNI data:

We included dMRI data of 216 subjects from the ADNI dataset (all ADNI phases) in our classification experiments. The data comprises three subgroups: 72 patients with diagnosed Alzheimer’s disease (AD), 72 patients with mild cognitive impairment (MCI), and 72 HC. The three groups were matched for age and sex. All datasets as well as further information about the data are accessible via https://ida.loni.usc.edu/. The ADNI was launched in 2003 as a public-private partnership, led by Principal Investigator Michael W. Weiner, MD. The primary goal of ADNI has been to test whether serial magnetic resonance imaging, other biological markers, and clinical and neuropsychological assessment can be combined to measure the progression of MCI and early AD. For up-to-date information, see www.adni-info.org. Data were acquired on GE and Siemens 3 T MRI scanners with a varying number of gradient directions between 16 and 48 at *b* = 1,000*s*/*mm*^−2^ and a varying anisotropic resolution between 1.0 and 2.7 mm. The IDs of all included subjects can be found in Supplementary S4 and the corresponding imaging parameters can be accessed via the dataset webpage.

The following ten tracts commonly associated with AD were included in our experiments on the ADNI dataset: Rostrum (CC_1), Genu (CC_2), Rostral Body (CC_3), Anterior Midbody (CC_4), Posterior Midbody (CC_5) and Isthmus (CC_6) of the Corpus Callosum (CC) as well as left and right Inferior Cerebellar Peduncle (ICP) and Superior Cerebellar Peduncle (SCP). Due to frequent errors in the TractSeg results in all datasets, the Splenium of the CC (CC_7) was excluded from the analysis (see Supplementary S1.4).

#### PPMI data:

We included dMRI data of 129 subjects from the PPMI dataset (baseline visit) in our classification experiments. The data comprises three subgroups: 43 patients with diagnosed Parkinson’s disease (PD), 43 patients with prodromal PD, and 43 HC. The three groups were matched for age and sex. All datasets as well as further information about the data are accessible via https://ida.loni.usc.edu/. Data were acquired on a Siemens 3 T MRI scanner with 64 directions with varying b-values of *b* = 600*s*/*mm*^−2^ and *b* = 1,000*s*/*mm*^−2^ as well as 2 mm isotropic resolution. The IDs of all included subjects can be found in Supplementary S4 and the corresponding imaging parameters can be accessed via the dataset webpage.

The following eight tracts commonly associated with PD were included in our experiments on the PPMI dataset: CC_1, CC_2, CC_3, CC_4, CC_5, CC_6 as well as left and right Corticospinal Tract (CST).

#### SCHZ data:

We included dMRI data of 98 subjects from the SCHZ dataset in our classification experiments. The data comprises two subgroups: 49 patients with diagnosed schizophrenia and 49 HC. The groups were matched for age and sex. This data was obtained from the OpenfMRI database (https://openfmri.org/dataset/ds000030/)^39^. Its accession number is ds000030. Data were acquired on a Siemens 3 T Tim Trio MRI scanner with 64 directions at *b* = 1,000*s*/*mm*^−2^ and 2 mm isotropic resolution. The IDs of all included subjects can be found in Supplementary S4 and the corresponding imaging parameters can be accessed via the dataset webpage.

The following fourteen tracts commonly associated with schizophrenia were included in our experiments on the SCHZ dataset: CC_1, CC_2, CC_3, CC_4, CC_5, CC_6 as well as left and right Thalamo-Prefrontal Tract (T_PREF), Thalamo-Parietal Tract (T_PAR), Striato-Parietal Tract (ST_PAR) and Striato-Fronto-Orbital Tract (ST_FO).

#### CAT data:

We included dMRI data of 87 subjects from the CAT dataset in our classification experiments. The data comprises three subgroups: 30 schizophrenia patients with catatonia, 29 schizophrenia patients without catatonia, and 28 HC. The groups were matched for age and sex. For the regression experiments, we used 59 schizophrenia patients from the CAT dataset and 49 additional schizophrenia patients which were not previously considered in the CAT analyses due to the lack of matching with HC (overall n = 108). The following demographic and clinical measures were included in our experiments:

- Age
- Pack-Years: the number of packs of cigarettes smoked per day by the number of years the person has smoked.
- Education: the number of years the person spent in an educational institution, such as high school or university.
- BPRS total: aggregated score on the Brief Psychiatric Rating Scale (BPRS), measuring the severity of various psychiatric symptoms.
- PANSS total: aggregated score on the Positive and Negative Syndrome Scale (PANSS), measuring symptom severity of patients with schizophrenia.
- GAF: score on the Global Assessment of Functioning scale, measuring the social, occupational, and psychological functioning of the person.
- OLZe: indicating the daily doses of antipsychotic medication in terms of Olanzapine equivalents (OLZe).

Data were acquired at CIMH on a Siemens 3 T Tim Trio MRI scanner with 60 directions at *b* = 1,500*s*/*mm*^−2^ and 1.7 mm isotropic resolution. The studies on the acquisition of CAT and HC data were approved by the local ethics committees (Medical Faculties Mannheim and Heidelberg at Heidelberg University, Germany). Written informed consent was obtained from all participants after a detailed explanation of the aims and procedures of the study. Further details about the dataset can be found in the work presented by Wasserthal and colleagues^27^.

The following fourteen tracts commonly associated with catatonia were included in our experiments on the CAT dataset: CC_1, CC_2, CC_3, CC_4, CC_5, CC_6 as well as left and right CST, ST_FO, Thalamo-Premotor Tract (T_PREM) and Striato-Premotor Tract (ST_PREM).

#### Data preprocessing and tract modeling:

The following artifact and noise correction steps were conducted for all dMRI images using MRtrix and FSL^42,43^: noise level estimation and denoising^44^, Gibbs ringing removal^45^, eddy current and inhomogeneity distortion correction^46,47^ as well as bias field correction^48^. The corrected images were then rigidly registered and resampled to the MNI-space FA template contained in the TractSeg package using MITK Diffusion. Brain masks were calculated using FSL Bet^49^. Tensors and FA maps were calculated and constrained spherical deconvolution (CSD) with a successive extraction of the three principal fiber directions (peaks) was performed using MRtrix^50^. The peaks served as input to TractSeg, which was used to calculate tract segmentations, tract start- and end-region segmentations, tract orientation maps, and as well as tractograms of each tract. A complete list of the used commands and parameters can be found in Supplementary S5.

### Tract Parcellation

The points of a statically resampled tract (see parcel assignment strategy (1) discussed in the introduction) can be seen as individual samples of a multi-class classification problem where the classes are not easily separable since the point clouds formed by the samples of the individual classes are partly overlapping. We propose to calculate the hyperplanes that optimally separate the point clouds with a minimal amount of wrong samples. These hyperplanes strictly divide 3D space into individual parcels while respecting splitting and fanning tract architectures. The task of finding such an optimal separation is solved by large margin classifiers.

**Figure 7:**
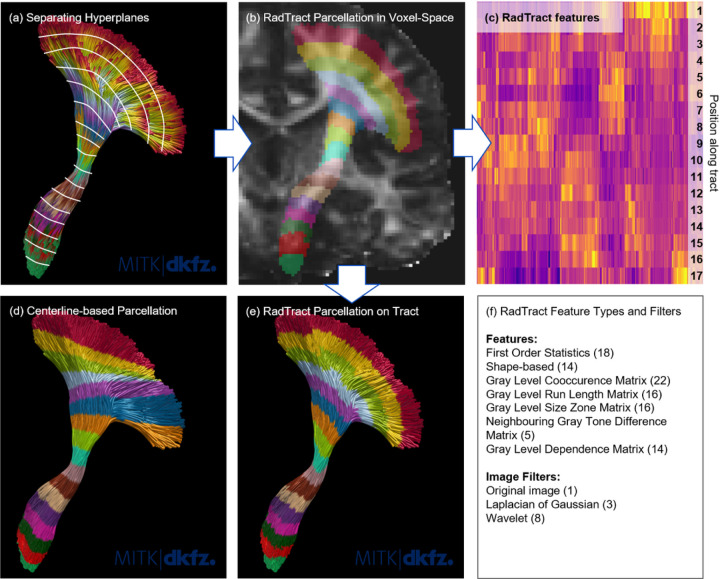
Illustration of the complete RadTract process. The points of a statically resampled tract (a) can be seen as samples of partly overlapping classes that are not linearly separable. We are aiming at finding the hyperplanes, superimposed as white lines on the tract in (a), that optimally separate the classes with the smallest amount of errors. This task can be solved using large-margin classifiers such as SVMs. This enables us to create parcellations directly in voxel-space (b) that do not suffer from projection-induced misassignments, as is the case in the centerline-based approach (d). For visualization purposes, the tract parcellation in voxel-space is projected back on the original streamlines (e). The proposed tract parcellation in voxel-space (b) is used to calculate 1106 features per parcel, visualized in (c). In the case of the CST example used in this figure, this results in 18,802 features for the complete tract. The calculated feature classes and used image filters are listed in (f).

RadTract implements this using a support vector machine (SVM), which belongs to the class of large margin classifiers, to assign each voxel along the tract to a specific parcel. For each tract, an SVM is trained on the fly using the point coordinates along the resampled streamlines as features and their position index as class label *l* ∈ [1… *n*]. To ensure short training times and to avoid a bias towards dense tract regions, the initial number of streamlines (here 10,000) is reduced to about 500 using the QuickBundles streamline clustering to merge similar streamlines^51^. The trained classifier is then applied to all voxel coordinates covered by the respective tract and the predicted label determines the voxel’s parcel. Figure 7 illustrates the complete parcellation process.

Consistent point ordering for the same tract across streamlines and subjects is ensured using a binary ROI delineating the start region of the tract, for example provided by TractSeg. Without start ROI, the intra-tract alignment of streamlines is achieved by reversing the point ordering of the streamlines that are flipped in terms of their minimum average flip distance (MDF) to the tract centerline^51^. In this case, inter-subject alignment can be achieved by using a single centerline across subjects.

While the number of parcels *n* can be defined arbitrarily, RadTract provides a method to automatically estimate the number of parcels from an input tract and image as

$$n_{auto}=\frac{n_{voxels}}{m}$$

1, where *n*_*voxels*_ is the average number of voxels traversed by the streamlines and *m* is the desired thickness of the tract parcels in tract direction. This ensures a roughly constant parcel size across tracts. In our experiments and as RadTract default, we chose *m* = 5 voxels, which ensures parcels that are large enough to avoid fragmentation and for calculating meaningful features.

### Feature Calculation

RadTract feature calculation is based on pyradiomics, a widely used open-source Python package for radiomics feature calculation^40^. The Feature calculation can be easily customized using yaml-based parameter files. The complete list of features used in this work can be found in Supplementary S6 and the yaml file used to parameterize the feature extraction is included in the supplementary code. Figure 7 (c) and (f) illustrates the calculated features as heatmap and provides a list of calculated feature types as well as the used image filters. To analyze the influence of individual feature classes on the experiment outcome, the complete feature set was further split into the following subsets:

1. All features, all filtered images
2. First-order features, all filtered images
3. Shape features, all filtered images
4. Gray level co-occurrence matrix features (GLCM), all filtered images
5. Gray level run length matrix features (GLRLM), all filtered images
6. Gray level size zone matrix features (GLSZM), all filtered images
7. Neighboring gray-tone difference matrix features (GLDM), all filtered images
8. Gray level dependence matrix features (NGTDM), all filtered images
9. All features, only unfiltered images
10. All features, LoG-filtered images
11. All features, wavelet-filtered images
12. First-order features, only unfiltered images

RadTract produces numerous features per tact, so automatic feature selection is vital for later analysis. A large variety of feature selection techniques exist and the best choice depends on the concrete task. A detailed analysis of this aspect would go beyond the scope of this work. In our experiments, we first removed all constant features as well as all highly correlated (Pearson correlation > 0.95) and therefore likely redundant features. Second, we used a simple and fast univariate feature selection implemented in scikit-learn on the training data to further automatically reduce the respective input feature set to 500 features^52^.

### Implementation Details

We used the support vector classification as well as random forest classification and regression implemented in scikit-learn (v1.1.2) in our implementation of the RadTract parcellation function as well as all classification and regression experiments^52^. Default parameterization was used if not stated otherwise. Pyradiomics v3.0.1 was used for all radiomics feature calculations^40^. Further used python packages include numpy (v1.23.3), scipy (v1.9.1), pydicom (v2.3.0), nibabel (v4.0.2), skimage (v0.19.3), dipy (v1.5.0), TractSeg (v2.7) and vtk (v9.2.0)^53–59^. Python version 3.10 was used in all experiments.

## Data Availability

The ADNI, PPMI, and SCHZ datasets are available via their respective websites. The CAT dataset is not publicly available.[1]

## Figures and Tables

**Figure 2:**
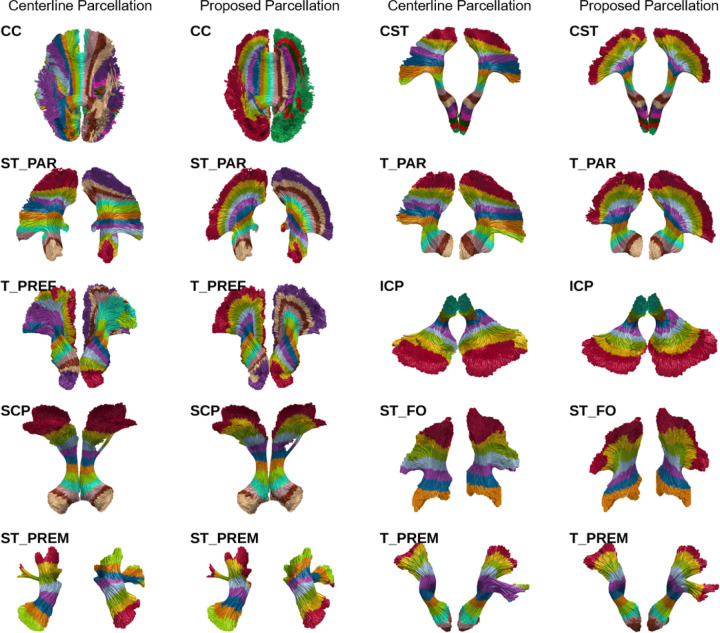
Illustration of the parcellation results using the centerline-based approach and proposed approach for one subject. It becomes apparent that, particularly in complex tract regions with a lot of fanning, RadTract produces parcellations with drastically improved anatomical cohesion. Due to space restrictions, the parcellation results of the complete corpus callosum are shown instead of the individual parts that were used for the subsequent experiments. The parcellations of the individual CC parts can be found in Supplementary S1.3.
